# Illness Integration in Caregivers’ Identity: Associations with Care-Burden, Well-Being, and Attachment Orientation

**DOI:** 10.1007/s10880-025-10110-3

**Published:** 2025-11-27

**Authors:** Karin Mashevich, Eva Bei, Shira Galin-Soibelman, Ofra Kalter-Leibovich, Tami Schifter, Noa Vilchinsky

**Affiliations:** 1 Bar-Ilan University, Ramat Gan, Israel; 2 University of Bologna, Bologna, Italy; 3 Bar-Ilan University, Ramat Gan, Israel; 4 The Gertner Institute, Sheba Medical Center, Ramat Gan, Israel, RAMAT-GAN, Israel

**Keywords:** Caregivers, Illness integration, Identity, Attachment, Care-burden

## Abstract

Caregiving can provide purpose and life satisfaction but is often linked to increased burden and reduced quality of life. Understanding mechanisms influencing caregivers’ experiences is crucial for effective support. One key factor is illness integration—the extent to which caregivers incorporate the patient’s illness into their identity. Caregivers may feel engulfed, reject, accept, or find enrichment in the illness, shaping their psychological outcomes. This study examines the relationships between illness integration, care burden, well-being, and attachment orientation to illuminate identity processes in caregiving and guide interventions to reduce strain and foster resilience. The results from our cross-sectional online survey of 162 informal caregivers indicated that caregivers predominantly accepted their loved ones’ illness into their identity, and this acceptance was in turn associated with both their well-being and care-burden. Caregivers high on attachment anxiety reported greater feelings of engulfment by the illness, leading to increased burden and diminished well-being. On the other hand, caregivers high on avoidant attachment struggled to accept the illness, and also experienced heightened burden. This study underscores the significance of illness integration in shaping caregivers’ experiences. The findings highlight the need to promote adaptive integration processes and address attachment-related challenges, reducing caregiver strain and enhancing resilience.

## Introduction

Informal caregivers, often family or friends, provide unpaid care and support while managing their own coping process (Revenson et al., 2016). They frequently experience emotional distress, burden, and heightened stress—sometimes exceeding that of patients (Revenson et al., 2016; Dekel et al., 2010).

Among patients (i.e., care recipients), illness affects socioeconomic status, relationships, mood (Shrestha et al., 2020; Brandt et al., 2022; Falvo & Holland, 2017) and disrupts the sense of self (Kafer, 2013). Oris et al. (2016) defined *illness identity* as the degree to which illness integrates into one’s identity, shaping perceptions, experiences, and daily functioning. Chronic illness alters self-perception (Charmaz, 1995), requiring adaptation, from rejecting illness to fully integrating it (Leventhal et al., 2020; Falvo & Holland, 2017; Van Bulck et al., 2019). This adaptation process has significant psychological and behavioral consequences (Carroll et al., 2020; Oris et al., 2016, (Oris, et al., 2018)). Oris et al. (2016) developed the Illness Identity Questionnaire (IIQ), assessing four states: *engulfment* (illness dominates identity), *rejection* (illness is psychologically distanced), *acceptance* (illness is acknowledged without defining self-worth), and *enrichment* (illness fosters growth, resilience, and meaning). Illness identity orientations influence well-being—acceptance is linked to lower distress, while engulfment correlates with greater distress (Van Bulck et al., 2019, Van Bulck, et al., 2018; Luyckx et al., 2018). Adler et al. (2021) found that patients who integrated illness without rejecting it had better well-being and coping.

Caregiving involves emotional and practical support (Schubart, 2014) and can foster resilience and gratitude (Quinn et al., 2015; Cheng et al., 2016; Quinn & Toms, 2019), but also heightens risks of anxiety, depression, and reduced well-being (Perpiñá-Galvañ et al., 2019; Yuan & Grühn, 2021). Caregivers often mirror patients’ distress (Missel et al., 2015), underscoring the dyadic nature of coping (Revenson et al., 2005; Traa et al., 2015; Karademas, 2022). Viewing illness as shared improves caregiver health and patient engagement (Gamarel et al., 2014; Krieger et al., 2015). Despite evidence of illness’s impact on both parties (Revenson et al., 2016; Cipolletta et al., 2020), research on the integration of the care recipient’s illness into the caregiver’s identity remains limited. This study aimed to examine such integration and its effects on caregiver well-being and burden.

Our second objective was to test whether caregivers’ illness integration is shaped by early developmental processes, particularly attachment orientation. Illness integration entails emotional and cognitive adjustments (Charmaz, 1995; Segal, 2018) rooted in coping styles formed in interpersonal contexts. Attachment, established in childhood, guides stress regulation (Bowlby, 1969; Mikulincer & Shaver, 2019): secure attachment fosters resilience, while insecure forms disrupt identity stability (Bendezú et al., 2019; Pittman et al., 2011). In caregiving, attachment orientation influences well-being and burden (George-Levi et al., 2020); secure caregivers report greater satisfaction and fulfilment (Kim et al., 2005, 2008; Karantzas et al., 2019), whereas insecure attachment predicts higher distress and burden (Kuscu et al., 2009; Dobson et al., 2022).

Avoidantly attached caregivers suppress their own needs, deactivating the attachment system under stress (Garrison et al., 2014). They may resist emotional closeness, finding caregiving burdensome and emotionally threatening (Shaver et al., 2019). Consequently, they are likely to reject integrating the patient’s illness into their identity. In contrast, anxiously attached caregivers hyperactivate the attachment system, becoming overwhelmed by the patient’s illness (Shaver & Mikulincer,^2010^). Their distress amplifies personal anxiety and an excessive need to provide care, even when unnecessary (Bassett & Aubé, 2013; Monin et al., 2013). Fearing abandonment, they may over-identify with the patient’s condition, leading to *engulfment* (George-Levi et al., 2017).

Attachment and identity are intertwined (Kerpelman & Pittman, 2018). Formed in infancy, attachment shapes self-perception and stress regulation (Bowlby, 1969), influencing identity stability during crises (Bayrak et al., 2018). We propose that attachment orientation shapes caregivers’ illness integration, ultimately impacting their caregiving experience.

We hypothesized as follows:

### Hypothesis 1

The positive dimensions of illness integration, namely acceptance and enrichment, would be positively associated with well-being and negatively with care-burden.

### Hypothesis 2

The negative dimensions of illness integration, namely engulfment and rejection, would be negatively associated with well-being and positively with care-burden.

### Hypothesis 3

Higher levels of anxious or avoidant attachment would be associated with lower well-being and higher care-burden.

### Hypothesis 4

The dimension of illness engulfment would mediate the link between anxious attachment and outcomes. Specifically, higher levels of anxious attachment would correlate with greater illness engulfment, leading to increased care-burden and decreased well-being.

### Hypothesis 5

The dimension of illness rejection would mediate the link between avoidant attachment and outcomes. Higher levels of avoidant attachment would correlate with greater illness rejection, leading to lower care-burden and increased well-being.

### Hypothesis 6

The dimension of illness acceptance and enrichment would mediate the link between insecure attachment and outcomes. Higher levels of avoidant and/or anxious attachment would associate with lower levels of acceptance and enrichment, leading to higher care-burden and decreased well-being.

## Method

### Participants and Procedure

We conducted an online survey on Camoni, a social network for individuals with chronic illnesses and their caregivers (Camoni, 2023). Caregivers aged 18+ were recruited through Qualtrics email invitations and homepage posts; no incentives or advertisements were used. Participation was voluntary with written informed consent. Ethical approval was obtained from the Gertner Institute Review Board (2 December 2019). Data were collected from January to March 2020 using a self-report questionnaire requiring complete responses, ensuring no missing data.

### Measures

*The Illness Identity Questionnaire* (Oris et al., 2016) consists of 25 statements rated on a 5-point Likert scale ranging from 1 (strongly disagree) to 5 (strongly agree), and has been found to be valid and reliable among adults with chronic illnesses (Oris et al., 2016; 2018). The IIQ includes five statements for rejection (e.g., “I refuse to see my illness as part of myself”; “I just avoid thinking about my illness”), five for acceptance (e.g., “I am able to place my illness in my life”; “I accept being a person with an illness”), eight for engulfment (e.g., “My illness completely consumes me”; “I am preoccupied with my illness”), and seven for enrichment (e.g., “Because of my illness, I have learned a lot about myself”; “Because of my illness, I know what I want out of life”). In the current study, the statements were rephrased to refer to the care recipient’s illness (i.e., the illness of the person for whom the participant provides care). Each participant received four scores, one for each dimension, calculated as the mean of the relevant items. The IIQ has been translated into Hebrew (Meyer and Lamash, 2021). In our sample, Cronbach’s α for rejection, engulfment, acceptance, and enrichment were 0.87, 0.71, 0.76, and 0.88, respectively.

*Experience in Close Relationships (ECR-RS; Fraley *et al., 2011). Caregivers’ attachment to the care recipient was assessed using the ECR-RS, in its Hebrew translation (K Shalem Research Center, n.d.). The ECR-RS is a self-report questionnaire designed to assess two dimensions of attachment: anxiety and avoidance. It includes nine items, three for the anxiety subscale (e.g., “I often worry that this person doesn’t really care for me”) and six for the avoidance subscale (e.g., “I prefer not to show a partner how I feel deep down”). Caregivers rate the extent to which each item describes their feelings toward their care recipient on a scale from 1 (not at all) to 7 (very much). Scores for each subscale are computed by averaging item responses. In our study, Cronbach’s α were 0.70 for anxious attachment and 0.74 for avoidant attachment.

*Care-Burden Inventory (CBI; Novak & Guest,* 1989). Care-burden was measured using the CBI, developed by Novak and Guest (1989). The CBI is a 24-item self-report questionnaire that assesses caregivers’ burden across various domains. Caregivers rate the extent to which each item describes their feelings on a 5-point Likert scale ranging from 1 (not at all) to 5 (very much), with higher scores indicating greater levels of burden. A total score is obtained by the sum of all item responses. Sample items include: “I don’t get a minute of rest,” “I feel that I am missing experiences in life,” and “I am angry about our relationship.” The Hebrew version was validated by Ben-Arzi et al. (2000). In our sample, Cronbach’s α was 0.95 for the complete scale.

*The Warwick-Edinburgh Mental Wellbeing Scale (WEMWBS; Tennant *et al., 2007). A 14-item self-report questionnaire was used to assess positive aspects of caregivers’ well-being, including positive affect, functioning, and satisfaction with interpersonal relationships. Caregivers selected the statement that best described their experience over the past two weeks using a 5-point Likert scale ranging from 1 (none of the time) to 5 (all of the time). A mean score (range 1–5) is obtained by summing all item responses, with higher scores reflecting greater well-being. Sample items include “I’ve been dealing with problems well” and “I’ve been feeling close to other people.” The questionnaire was translated into Hebrew and validated (Goodstein, 2012). In our sample, Cronbach’s α was 0.90.

### Data Analysis

We conducted checks of descriptive statistics to identify out-of-range values, outlier screening using z-scores (± 3.29 SD), and visual assessments of normality via histograms and Q-Q plots. Levene’s test verified homogeneity of variance for *t*-tests and ANOVAs. We assessed multicollinearity in regression and mediation models using Variance Inflation Factor to detect multicollinearity, with all values below 5.

For the descriptive analyses, and to test hypotheses 1–3, means and standard deviations (SDs) were computed for all study variables. To examine associations between sociodemographic characteristics and outcomes, we conducted correlation analyses (for age), independent-samples *t*-tests (for gender), and analyses of variance (ANOVAs) (for education, relationship status, socioeconomic status, religiosity, caregiver’s health condition, relationship to the care recipient, and care recipient’s health condition). Post hoc analyses, using Tukey tests, were planned and conducted to explore differences between subgroups where significant group effects were identified. In constructing the regression models, we included only the sociodemographic variables that showed significant associations with the outcomes in the preliminary analysis.

To examine mean differences among the four illness identity dimensions (acceptance, rejection, enrichment, and engulfment), we conducted a repeated measures within-subject ANOVA, as each participant contributed scores on all dimensions. This approach allowed us to test whether certain identity dimensions were endorsed more strongly than others within the same individuals, providing a direct comparison across the four dimensions.

Finally, to test hypotheses 4–6, we constructed regression models including all sociodemographic variables that were significantly associated with these outcomes as covariates (see Results: Participants and Preliminary Analysis). We then tested four mediation models using Preacher and Hayes’ (2004) bootstrapping procedure: (1) anxious attachment predicting well-being, (2) avoidant attachment predicting well-being, (3) anxious attachment predicting care-burden, and (4) avoidant attachment predicting care-burden. In each model, the four illness integration factors served as potential mediators.

## Results

### Participants and Preliminary Analysis

The sample consisted of 162 caregivers (M age = 57.4 years, SD = 15.2), with 67.3% identifying as women and 32.7% as men. Educational attainment was varied: 34.6% held at least a bachelor’s degree, 24.1% had a master’s or PhD, while 20.4% reported post-secondary vocational training, 17.9% had completed secondary education, and 3% reported other forms of education. Most participants were married or partnered (75.9%), with the remainder being single (10.5%), divorced (9.9%), or widowed (3.7%). Socioeconomic status was self-reported as below average by 40.8%, average by 29.6%, and above average by 29.6%; “average” level was determined based on government household income data from the time of data collection. Regarding religiosity, 70.4% identified as secular, 16% as traditional, and 13.6% as religious. In this Israeli classification, “traditional” refers to a lower level of observance than “religious,” representing points on a continuum of religiosity. Nearly half of the caregivers (45.1%) reported a physical impairment or disability, 11.1% had multimorbidity, 6.8% had a mental health condition, and 37% reported no health conditions. Most participants cared for a spouse or partner (41.4%), followed by a parent (27.2%), child (17.8%), another family member (7.4%), or a non-relative (6.2%). Common care recipient conditions included cancer or heart disease (34%), multimorbidity (26.5%), and physical disabilities (13%), with fewer cases of Alzheimer’s or memory impairment (8.6%), aging-related issues (4.9%), mental health conditions (4.4%), or other illnesses (8.6%).

Notably, caregiver health status, care recipient health condition, and relationship type were significantly associated with care-burden, as shown in the ANOVA results presented in Table 1. Caregivers with multiple health conditions reported higher burden than those without, and those caring for a spouse/partner, parent, or child reported higher burden than those caring for a non-family member. Although recipient medical status was associated with care-burden, post hoc tests did not find significant differences between specific conditions. For well-being, significant associations emerged with age, gender, and caregiver-recipient relationship. Age correlated positively with well-being (*r* =.21, *p* <.01), and partnered or divorced caregivers reported higher well-being than single caregivers. Additionally, caring for a parent or child was linked to lower well-being compared to caring for a non-family member.

**Table 1:** Differences in well-being and care-burden depending on sociodemographic variables

Variable		Care-burden				Well-being		
		*N*	*M* (*SD*)	tor F*	*p* value	*M* (*SD*)	t or F*	*p* value
Gender	Female	53	34.82 (23.02(	− 1.49	.13	3.35 (1.33)	2.16*	.03
	Male	109	29.26 (23.37)			3.62 (1.78)		
Education	Secondary	29	41.96 (22.21)	1.74	.14	3.28 (1.33)	.75	.533
	Vocational education	33	3.633 (21.85)			3.45 (0.90)		
	Bachelor’s degree	56	31.19 (21.50)			3.47 (0.57)		
	Master’s/PhD	39	28.94 (21.15)			3.51 (0.60)		
	Other	5	28.80 (20.81)			3.06 (0.94)		
Relationship Status	Single	17	40.47 (22.21)	1.11	.34	2.95 (1.33)	3.01*	.03
	Partnered	123	31.32 (21.85)			3.47 (0.89)		
	Divorced	16	36.75 (21.50)			3.63 (0.57)		
	Widowed	6	36.33 (21.15)			3.64 (0.60)		
Socio-economic Status	Below average	66	37.04 (22.21)	2.12	.12	3.38 (1.33)	2.75	.06
	Average	48	31.56 (21.85)			3.30 (0.89)		
	Above average	48	28.89 (21.50)			3.64 (0.57)		
Religiosity	Secular	114	32.51 (22.21)	.582	.56	3.39 (1.33)	4.46*	.01
	Traditional	26	31.30 (21.85)			3.82 (0.89)		
	Religious	22	37.54 (21.50)			3.24 (0.57)		
Caregiver’s Health Condition	Physical impairment or disability	73	33.41 (23.11)	3.11*	.02	3.47 (1.33)	1.31	.27
	Mental health	11	35.36 (22.78)			3.32 (0.91)		
	Multi-morbidity	18	45.61 (22.46)			3.13 (0.63)		
	No conditions or disabilities	60	28.30 (23.44)			3.51 (1.78)		

Variable		Care-burden			Well-being			
		N	*M* (*SD*)	t or F*	*p* value	*M* (*SD*)	t or F*	*p* value
Relationship to the Care Recipient	Spouse or Partner	67	31.97 (22.21)	4.76**	.00	3.53 (1.33)	3.30*	.01
	Parent	44	39.25 (21.88)			3.25 (0.89)		
	Child	29	36.55 (21.53)			3.25 (0.57)		
	Another family member	12	27.25 (21.18)			3.53 (0.60)		
	Nonrelative	10	9.10 (20.84)			4.07 (0.94)		
Care Recipient’s Health Condition	Alzheimer’s disease or any other serious memory impairment	14	4300. (22.21)	2.531*	.02	3.09 (1.33)	1.44	.20
	Aging	8	31.80 (21.85			3.27 (0.89)		
	Physical impairment or disability	21	27.63 (21.50)			3.63 (0.57)		
	Cancer or heart disease	55	49.85 (21.15)			3.50 (0.60)		
	Mental health	7	28.87 (20.81)			3.51 (0.94)		
	Multi-morbidity	43	37.62 (20.47)			3.41 (1.38)		
	Other	14	25.64 (20.15(			3.25 (1.86)		

This table presents *t*-tests (t), analysis of variance tests (*F*) and correlations, conducted to view the associations between sociodemographic characteristics and outcome variables. *p* <.05*. *p* <.01**. *N* = Number of participants

Table 2 presents the means, standard deviations, and intercorrelations among key study variables. The analysis revealed significant relationships between illness identity dimensions and caregiver outcomes.

**Table 2:** Means, SDs, and intercorrelations between care-burden, well-being, attachment anxiety, attachment avoidance, and illness identity dimensions

Variable	Mean (SD)	Min	Max	*R*							
				(1)	(2)	(3)	(4)	(5)	(6)	(7)	(8)
(1) Care-burden	33 (21.78)	.00	89								
(2) Well-being	3.44 (.75)	1.38	5	−.55**							
(3) Anxious attachment	3.83 (1.47)	1	7	.48**	−.38**						
(4) Avoidant attachment	2.11 (1.53)	1	7	.38**	−.22**	.23**					
(5) Rejection	2.23 (.92)	1	4.6	−.13	−.02	−.01	−.08				
(6) Engulfment	2.97 (.99)	1	5	.52*	−.48**	.26**	.15*	−.02			
(7) Acceptance	3.48 (1.03)	1	5	−.35**	.17*	−.15	−.29**	−.09	.09		
(8) Enrichment	3.32 (1.09)	1	5	.19	.16*	−.13	.02	.03	.19*	.18*	

This table presents the means, SDs, and associations between the study variables, which include illness identity dimensions, attachment orientations, and outcome variables. *p <.05. **p <.01

The repeated measures within-subject ANOVA confirmed significant differences among the four illness identity dimensions (*F* = *52.62, p* < *.01*). Acceptance had the highest mean (*M* = *3.48*), significantly exceeding rejection (*M* = *2.23*) and engulfment (*M* = *2.97*; *p* < *.01*), while rejection had the lowest mean (*p* <.01). Enrichment (*M* = *3.32*) scored higher than rejection and engulfment (*p* <.01) but did not surpass acceptance.

### Mediation Analysis

**Fig. 1:**
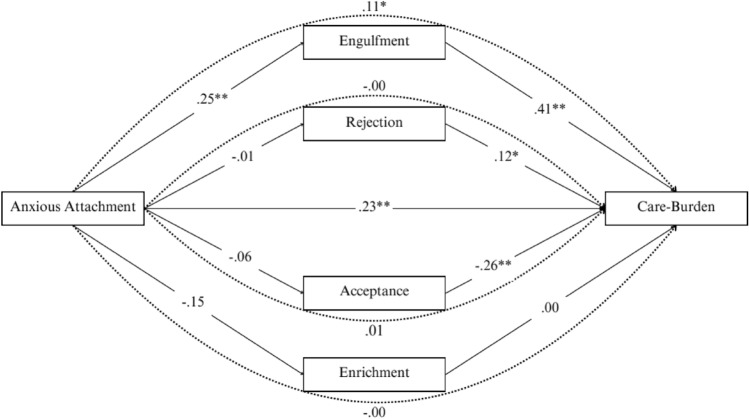
The direct and indirect effects (standardized coefficients) of the illness identity dimensions on the association between anxious attachment and care-burden. *Note.* The straight lines represent direct associations, and the dotted lines represent indirect associations. Percentile of bootstrapping 95% confidence interval, the number of bootstrap samples = 5000. *p <.05. ** *p* <.01

The first mediation analysis was performed to assess the mediating role of illness identity dimensions in the relation between *anxious attachment* and care-burden. Significant demographic variables identified from the ANOVAs related to care-burden were included as dummy variables in this model to control for their effects. Avoidant attachment was also controlled for in this model. The total effect of anxious attachment on care-burden was significant (*β* = *6.19, p* < *.01*). With the inclusion of the four illness identity dimensions, the direct effect of anxious attachment on care-burden remained significant. A positive direct association was found between anxious attachment and engulfment (*β* = *4.42, p* < *.01*), with engulfment also serving as a significant mediator. The indirect effect of anxious attachment on care-burden through engulfment was significant (*indirect-effect* = 0.11, *BootSE* = 0.03, 95% CI [0.04, 0.18]). There were no significant direct effects between the other illness identity dimensions (i.e., rejection, enrichment, and acceptance) and anxious attachment. Specifically, the indirect effects for rejection (*indirect effect* = − 0.00, *BootSE* = 0.01, 95% CI [− 0.02, 0.02]), enrichment (*indirect-effect* = − 0.00, *BootSE* = 0.01, 95% CI [− 0.02, 0.02]), and acceptance (*indirect-effect* = 0.01, *BootSE* = 0.02, 95% CI [− 0.02, 0.06]) were non-significant. These results demonstrate that engulfment partially mediates the association between anxious attachment and care-burden: The higher the anxious attachment, the higher the engulfment score, which was also associated with higher care-burden (see Fig. 1).

**Fig. 2:**
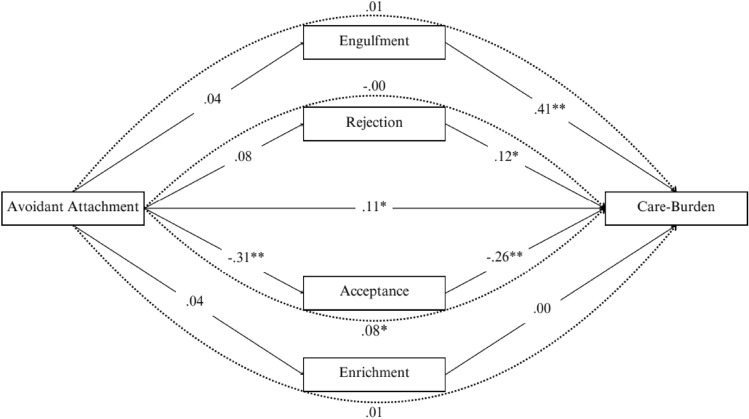
The direct and indirect effects (standardized coefficients) of the illness identity dimensions on the association between avoidant attachment and care-burden. *Note.* The straight lines represent direct associations, and the dotted lines represent indirect associations. Percentile of bootstrapping 95% confidence interval, the number of bootstrap samples = 5000. *p <.05. **p <.01

The second mediation analysis was performed to assess the mediating role of illness identity dimensions in the relation between avoidant attachment and care-burden. Significant demographic variables identified from the ANOVAs related to care-burden were included as dummy variables in this model to control for their effects. Anxious attachment was also controlled for in this model. The total effect of avoidant attachment on care-burden was significant (*β* = 0.22, *p* <.01). With the inclusion of the four illness identity dimensions, the effect of avoidant attachment on care-burden remained significant (*β* = 0.12, *p* =.05). A direct negative association was found between avoidant attachment and acceptance (*β* = − 0.31, *p* <.01), with acceptance also serving as a significant mediator. The indirect effect of avoidant attachment on care-burden through acceptance was significant (*indirect effect* = 0.08, *BootSE* = 0.03, 95% CI [0.02, 0.15]). There were no significant effects between avoidant attachment and the other illness identity dimensions. Specifically, the indirect effects for engulfment (*indirect effect* = 0.01, *BootSE* = 0.03, 95% CI [− 0.04, 0.09]), rejection (*indirect-effect* = 0.00, *BootSE* = 0.00, 95% CI [− 0.01, 0.01]), and enrichment (*indirect-effect* = 0.01, *BootSE* = 0.01, 95% CI [− 0.01, 0.04]) were non-significant. These findings demonstrate that acceptance partially mediated the association between avoidant attachment and care-burden: The higher the avoidant attachment, the lower the acceptance score, which was associated with higher care-burden scores (see Fig. 2).

**Fig. 3:**
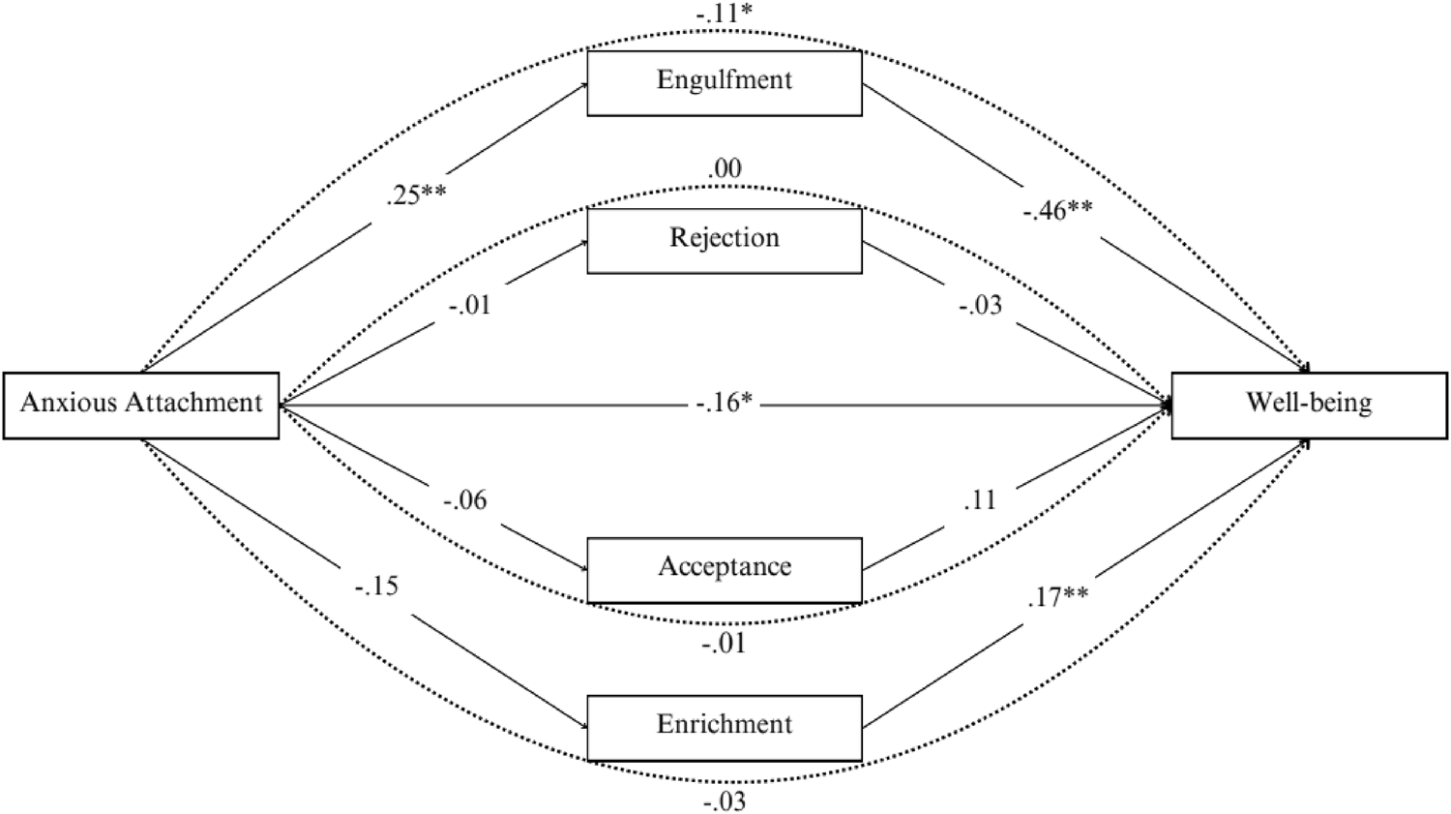
The direct and indirect effects (standardized coefficients) of the illness identity dimensions on the association between anxious attachment and well-being. *Note.* The straight lines represent direct associations, and the dotted lines represent indirect associations. Percentile of bootstrapping 95% confidence interval, the number of bootstrap samples = 5000. *p <.05. **p <.01

The third mediation analysis was performed to assess the mediating role of illness identity dimensions in the relation between anxious attachment and well-being. Significant demographic variables identified from the ANOVAs related to well-being were included as dummy variables in this model to control for their effects. Avoidant attachment was also controlled for in this model. The total effect of anxious attachment on well-being was significant (*β* = − 0.31, *p* <.01). With the inclusion of the four illness identity dimensions, the direct effect of anxious attachment on well-being remained significant (*β* = − 0.16, *p* <.05). Engulfment was identified as a mediator in the association between anxious attachment and well-being, with a significant indirect effect (*indirect-effect* = − 0.11, *BootSE* = 0.04, 95% CI [− 0.20, − 0.03]). There were no significant effects between the other illness identity dimensions and anxious attachment. Specifically, the indirect effects for rejection (*indirect-effect* = − 0.02, *BootSE* = 0.02, 95% CI [− 0.06, 0.00]), acceptance (*indirect-effect* = − 0.00, *BootSE* = 0.01, 95% CI [− 0.03, 0.01]), and enrichment (*indirect-effect* = 0.00, *BootSE* = 0.00, 95% CI [− 0.01, 0.01]) were non-significant. These findings demonstrate that engulfment partially mediated the association between anxious attachment and well-being: The higher the anxious attachment, the higher the engulfment score, which was associated with lower well-being scores (see Fig. 3).

The fourth mediation analysis was performed to assess the mediating role of illness identity dimensions in the relation between avoidant attachment and well-being. Significant demographic variables identified from the ANOVAs related to well-being were included as dummy variables in this model to control for their effects. Anxious attachment was also controlled for in this model. The total effect of avoidant attachment on well-being was significant (*β* = − 0.15, *p* <.05). With the inclusion of the four illness identity dimensions, the direct effect of avoidant attachment on well-being remained significant (*β* = − 0.09, *p* >.05). None of the illness identity dimensions mediated this relationship, as all indirect effects were non-significant. Specifically, the indirect effects for engulfment (*indirect-effect* = − 0.03, *BootSE* = 0.03, 95% CI [− 0.10, 0.03]), rejection (*indirect-effect* = 0.00, *BootSE* = 0.01, 95% CI [− 0.02, 0.03]), acceptance (*indirect-effect* = − 0.03, *BootSE* = 0.02, 95% CI [− 0.08, 0.00]), and enrichment (*indirect-effect* = 0.00, *BootSE* = 0.01, 95% CI [− 0.02, 0.01]) were non-significant. These findings indicate that no mediation effects were observed between avoidant attachment and well-being through the illness identity dimensions.

## Discussion

### Illness Identity and Caregivers’ Psychological Health

We examined how caregivers integrate the illness of their loved ones into their identity and its impact on psychological health. Findings indicate that caregivers do incorporate the illness into their identity, significantly shaping their experiences. Consistent with prior research, illness integration affects caregivers’ identity, caregiving experience, and quality of life (Anderson & White, 2018; Eifert et al., 2015; Bassi et al., 2016).

Caregivers with higher illness *acceptance* reported lower care-burden, but acceptance was not linked to overall well-being. Acceptance likely reduces burden by allowing caregivers to integrate illness into their identity without letting it dominate other life aspects (Oris et al., 2016; Benson et al., 2015). However, while acceptance aids caregiving demands, it may not improve well-being outside of caregiving, as the illness remains a pervasive stressor (Brandt et al., 2022). Thus, Hypothesis I was only partially supported: acceptance was associated with lower care-burden but not with higher well-being.

Conversely, caregivers who experienced *enrichment* reported enhanced well-being, likely due to personal growth, strengthened relationships, and a greater sense of purpose (Reeve & Lincoln, 2002; Tedeschi & Calhoun, 2004; Helgeson et al., 2006). This finding partially supports Hypothesis 1, as enrichment was associated with higher well-being but showed no associations with care-burden, suggesting that while it fosters well-being, it does not mitigate caregiving’s practical burdens (Senol-Durak, 2014).

*Rejection* of illness correlated with higher care-burden but did not affect well-being, whereas *engulfment* was associated with both increased care-burden and lower well-being.

These results partially support Hypothesis 2: while rejection was related to greater care-burden but not to well-being, engulfment was consistently linked to both higher care-burden and lower well-being. These findings align with research showing that caregivers who lose their sense of individual identity due to their caregiving role experience greater distress (Aubeeluck & Buchanan, 2006; Kayaalp et al., 2021). Caregivers engulfed by illness often perceive it as consuming their lives, diminishing well-being even outside caregiving (Aubeeluck & Moskowitz, 2008).

### Illness Identity, Attachment Orientation, and Caregiving Experience

Anxious attachment was linked to lower well-being and higher care-burden (Nicholls et al., 2014; Karantzas et al., 2019; Romano et al., 2022). This finding supports Hypothesis 3, as anxious attachment was associated with both reduced well-being and increased care-burden. Moreover, the mediation by engulfment supports Hypothesis 4, indicating that anxious attachment was associated with higher levels of engulfment, which in turn were associated with both greater burden and greater distress. It was not associated with positive illness integration but was mediated by *engulfment*, which heightened burden and distress. These findings align with research showing that negative events hyperactivate the attachment system, increasing distress and impairing emotion regulation (Mikulincer & Shaver, 2019; George-Levi et al., 2020). Engulfment appears to amplify this cycle, reinforcing caregiving stress (Bowlby, 1977 George-Levi et al., 2017).

Attachment avoidance was associated with greater care-burden and lower well-being (Li & Fung, 2014; Lan et al., 2021; Romano et al., 2022). Although this is consistent with the general pattern hypothesized in Hypothesis 3, Hypothesis 5 was not supported, as illness rejection did not mediate the association between avoidant attachment and care experience (i.e., care-burden and well-being. While avoidantly attached individuals often cope by distancing themselves from stressors , rejection did not mediate this association. This suggests that avoidance is less effective in the caregiving context, where active involvement is required (Dixe et al., 2019; Dombestein et al., 2020). Previous studies indicate that avoidant attachment strategies are ineffective in coping with illness-related distress ( Karantzas et al., 2019; Marrero-Quevedo et al., 2019; Hasson-Ohayon et al., 2015).

Overall, attachment orientation plays a key role in shaping caregiving experiences. Anxiously attached caregivers struggle to maintain boundaries, leading to engulfment and distress, whereas avoidantly attached caregivers resist integration, limiting the potential benefits of acceptance.

### Attachment, Identity Development, and Stressors

Attachment and identity development are deeply intertwined (Kerpelman & Pittman, 2018). Insecure attachment hinders adaptive identity formation by disrupting the integration of personal and relational experiences into a coherent self-concept (Handa & Umemura, 2023). The current findings extend this understanding, showing that insecure attachment also impairs the adaptive integration of illness into identity. When early attachment is insecure, adulthood stressors—such as caring for an ill loved one—are more likely to be integrated in maladaptive ways, reinforcing distress. This interpretation supports Hypothesis 6, as insecure attachment was linked to lower levels of adaptive illness integration, contributing to greater burden and diminished well-being.

### Limitations and Future Directions

This study’s cross-sectional design limits causal inferences. However, given attachment’s stability across adulthood (Stevenson et al., 2019), a causal relationship between attachment and illness identity integration is plausible. A larger, more homogeneous sample would improve representativeness, as caregiver dynamics and illness characteristics vary widely. Additionally, future research should consider illness severity, time since diagnosis, and duration of caregiving, as these factors may influence caregivers’ experiences (Rolland, 1994).

### Implications for Healthcare Systems

Despite recent shifts (Kokorelias et al., 2019), healthcare systems remain largely patient-focused, often overlooking caregiver well-being. Our findings highlight that caregivers integrate the illness into their identity, affecting their mental health and caregiving experience. A holistic approach that includes caregiver support—addressing attachment patterns, illness identity dimensions, and stress management—could reduce burden and enhance well-being, ultimately benefiting both caregivers and patients.

The current findings suggest that illness identity questionnaires may serve as valuable screening tools for identifying vulnerable patients and caregivers within clinical settings. Moreover, they underscore the importance of understanding illness as a multidimensional experience—not only for patients but also for their caregivers—encompassing psychological, relational, and identity-related dimensions. Finally, adopting a dyadic approach that views patients and caregivers as an interconnected unit may significantly enhance the quality of clinical care (Traa et al., 2015).

## Data Availability

The datasets generated and analyzed during the current study are publicly available. The data were registered and can be accessed via the Open Science Framework (OSF) at the following link:https://osf.io/rm9cb/
